# Accessing Germany’s Electronic Patient Record Through Health Insurer–Provided Apps: Qualitative Interview Study

**DOI:** 10.2196/95294

**Published:** 2026-09-14

**Authors:** Susann May, Manuela Marquardt, Gina Barzen, Frances Seifert, Mary Nyarko, Antonia Niemeyer, Sonja Gscheidle, Liane Schenk, Sebastian Spethmann, Felix Muehlensiepen

**Affiliations:** 1 German Heart Center at Charité – University Medical Center Berlin, corporate member of Freie Universität Berlin and Humboldt-Universität zu Berlin Berlin, Berlin Germany; 2 User Experience in Digital Health Lab, Center for Health Services Research, Brandenburg Medical School Theodor Fontane Rüdersdorf Germany; 3 Institute of Medical Sociology and Rehabilitation Science Charité - Universitätsmedizin Berlin Berlin, Berlin Germany; 4 DZHK (German Centre for Cardiovascular Research) Berlin Germany

**Keywords:** accessibility, digital health, EHR, electronic health record, health, patient empowerment, qualitative interviews

## Abstract

**Background:**

Nationwide electronic health records (EHRs) are intended to strengthen patient empowerment and improve information continuity across health care settings. In Germany, the electronic patient record (elektronische Patientenakte [ePA]) transitioned from an opt-in to an opt-out model in 2025. Initial survey data suggest that technical complexity and access requirements may hinder engagement; yet, little is known about how users experience this access process.

**Objective:**

This study explored users’ experiences of setting up and authenticating access to insurer-provided ePA apps and examined the barriers affecting successful access.

**Methods:**

We conducted a qualitative interview study with individuals covered by statutory health insurance. From 61 interviews conducted within the broader ePA4all project, 23 were selected because participants had attempted to access the ePA through an insurer-provided application and described at least 1 concrete action or event during setup or authentication. Semistructured telephone interviews were conducted between August and December 2025. Data were analyzed using Kuckartz’s structured qualitative content analysis. The process phases were developed inductively from the material. All 23 interviews were independently coded by 2 researchers, with differences resolved through consensus. A second analytic step examined how barriers interacted and accumulated across the access pathway.

**Results:**

The sample included 21 participants who had successfully accessed the ePA and 2 who had initiated but abandoned setup or authentication. Setup and authentication were experienced not as a single technical step but as an interdependent process comprising 5 phases: orientation, technical entry, verification, interruption, and transition to initial use. During orientation, incomplete or outdated information left requirements and procedural steps unclear. Technical entry required users to coordinate different apps, interfaces, and device requirements. Verification involved multiple credentials, identification services, and partly repeated security steps. Technical failures, administrative inconsistencies, postal procedures, and waiting periods interrupted progression and sometimes required users to repeat previously completed steps. After successful access, an unclear immediate benefit could limit the transition to initial use. Across phases, participants relied on digital competence, prior experience, persistence, and self-efficacy to navigate the process. Difficulties were also reported by participants with high affinity for technology. Barriers could accumulate across phases: insufficient orientation increased subsequent coordination work, failures produced interruptions and repeated attempts, and prolonged effort contributed to frustration or disengagement.

**Conclusions:**

Access to the opt-out ePA through insurer-provided apps is shaped by cumulative demands rather than by isolated technical problems. Formal provision of an EHR does not ensure that users can successfully complete the access process. Implementation should prioritize clear preregistration guidance, continuity across applications and identification services, resumable procedures, actionable error messages, and accessible support. More broadly, onboarding and authentication should be treated as integral stages in the implementation of patient-facing digital health infrastructures rather than as neutral technical prerequisites.

**Trial Registration:**

German Clinical Trials Register DRKS00037053; https://www.drks.de/search/de/trial/DRKS00037053

**International Registered Report Identifier (IRRID):**

RR2-10.1177/20552076251407130

## Introduction

As part of the digital transformation within health care systems, implementation of patient-centered health technologies into treatment pathways is increasing. These developments include the global introduction of nationwide electronic health records (EHRs) aimed at empowering individuals to manage their own health and health data more competently. EHRs are intended to improve the availability of health information to patients and encourage their active participation in care processes [1], thereby contributing to improving the quality of medical care and helping health care systems meet future challenges [1]. Patient empowerment constitutes a central guiding principle of these digital health initiatives, aiming to strengthen patients’ self-efficacy and confidence in managing their health and navigating interactions with health care professionals (HCPs) [1-4].

Despite these intended benefits, the acceptance and active use of national EHR systems cannot be taken for granted. Data protection, data security, and trust in the transparent handling of health data are key determinants of the acceptance or rejection of national EHR systems [5,6]. Concerns about unauthorized access and data breaches represent some of the most frequently reported barriers [7,8]. Furthermore, usability challenges, such as complex interfaces, unclear navigation, and limited perceived value or benefit, reduce individuals’ motivation to use digital health records [9].

International evidence from patient portals indicates that formal availability does not automatically translate into successful enrollment and access. In a survey of patients who had not enrolled in a portal, almost two-thirds had not attempted enrollment, most commonly because of insufficient information or motivation; negative attitudes and computer-related obstacles were additional reasons [10]. A qualitative study of the US Veterans Health Administration’s national patient portal further showed that its multistep enrollment pathway, including online registration, in-person identity verification, and additional activation steps, created administrative and logistical barriers for patients [11]. These findings suggest that onboarding and authentication are not merely preliminary technical tasks but implementation stages that can determine whether patient-facing digital health infrastructures are reached in practice. However, the interaction and accumulation of barriers across these stages remain insufficiently understood.

In Germany, the electronic patient record (elektronische Patientenakte [ePA]) was introduced in 2021 [12]. At that time, individuals who had statutory health insurance could decide whether they wanted to apply for and use an ePA provided by their respective health insurance fund. In January 2025, the system transitioned from an opt-in to an opt-out model [13]. Since then, an ePA has been automatically created for all individuals covered by statutory health insurance, with the aim of enabling the storage and exchange of medical documents, facilitating cross-sectoral information flow, and strengthening patients’ roles within the care process [14]. Although the ePA is established automatically, its use remains voluntary. Individuals who do not wish to use the ePA may actively object to its use. Given the central role of statutory health insurance within the German health system, this regulatory change has broad practical relevance. Approximately 75 million of Germany’s 83.5 million inhabitants are covered by statutory health insurance, and 93 independent statutory health insurance funds (as of January 2026) [15] operate autonomously within a common legal framework [16]. Each statutory health insurer provides its members with its own ePA application. Access to the ePA is provided through apps offered by the respective statutory health insurers. Consequently, the ePA constitutes a national infrastructure, whereas the interfaces and parts of the setup process are implemented through multiple insurer-provided apps. While functional and technical requirements are defined by law and embedded within the national telematics infrastructure [17], differences remain in interface design, user guidance, and the specific implementation of the registration process. Consequently, access to the ePA may vary depending on the respective health insurer.

Initial findings on the use of the ePA indicate that it has thus far been used only to a limited extent. In a survey conducted in 2025, a total of 1500 individuals covered by statutory health insurance were asked about their use of the ePA. Twelve percent of respondents reported having actively set up their ePA and used it at least once. In contrast, 79% were classified as passive users who had not independently accessed their ePA to date, while 7% stated that they had objected to its use [18]. Passive users were further queried about their reasons for nonuse. Eleven percent selected the following response: too complicated, inconvenient, application does not work, no/old mobile phone [18]. These findings suggest that the uptake of the ePA may, at least in part, be hindered by challenges related to access requirements and technical prerequisites. In particular, these findings point to the importance of the initial setup and authentication process as a potential barrier to use. However, this process has so far received little systematic attention.

The setup of the ePA application is a complex process involving multiple technical and organizational requirements. In terms of actor-network theory, ePA authentication can be conceptualized as an obligatory passage point (OPP) [19]: as a necessary bottleneck, it bundles the interactions of heterogeneous actors (patient, smartphone, ID card, electronic ID [eID], application ecosystem, network connection, and backend infrastructure) and makes access to the ePA dependent on these elements being successfully aligned in a multistage sequence. A disruption in one component may therefore interrupt the entire access pathway, even when all other requirements have been met. From this perspective, authentication requires users not only to operate an application but also to coordinate technical systems, identification procedures, and organizational requirements. From this perspective, access to the ePA is not a simple technical step but a sociotechnical process that may generate barriers at different stages. Despite this conceptualization, empirical research has so far paid little attention to how users actually experience this process.

To date, there have been no studies on the subjective experiences of individuals during the ePA application setup process, mainly due to the recent introduction of the opt-out model in Germany. As a result, there is currently little insight into how users experience the registration process or at which specific stages problems may arise. Previous studies have primarily focused on the usability of digital health technologies themselves rather than on the processes of accessing or setting up these systems [8,20]. However, the effective use of the ePA presupposes that access to the ePA application is both available and manageable for users. Against this background, a deeper understanding of user experiences across the entire registration process is essential. By systematically examining challenges along this process, critical points that may hinder successful access can be identified. This study therefore explored how individuals with statutory health insurance experienced setup and authentication for their insurer-provided ePA application and which barriers affected successful access to the ePA. Initial use after authentication was considered only insofar as participants described the immediate transition from formal access to engagement with the application.

## Methods

### Ethical Considerations

All ethical issues were addressed. All study procedures were approved by the responsible ethics committee. All methods were performed in accordance with relevant guidelines and regulations. This study involves human participants and was approved by the Ethics Committee of the Charité Berlin (reference ID: EA1/333/24). Participants were informed verbally and in writing about the purpose, procedure, and significance of the study, as well as the associated benefits and risks, and were given the opportunity to ask questions. They were also informed that they had the right to withdraw their consent to participate in the study at any time, either verbally or in writing, without giving reasons. They were also informed that personal data would be collected and stored, whereby the data would be anonymized, but no data would be published that could be used to identify the individual. For security reasons, the data received from participants were always stored in a password-protected folder on a secure desktop computer. Written consent was obtained after participants had the opportunity to ask questions. Patients were not involved in the design of this study. Participants were offered €50 (approximately US $59.48) as an incentive for their participation in the study.

### Overview

The aim of the study was to investigate how individuals with statutory health insurance experienced setup and authentication for their insurer-provided ePA application in Germany. The study focused on the process from the users’ perspective, including perceived barriers and challenges from initial orientation to successful access to the ePA. Experiences after authentication were included only when they concerned the immediate transition to initial use. This study is part of the ePA4all project [21], which examines facilitators and barriers to the adoption of the ePA in Germany.

### Accessing the ePA Through Insurer-Provided Applications

Active access to the ePA is primarily provided through an application offered by the individual’s statutory health insurer and requires a multistep setup and authentication process. Alternatively, access via a stationary device (desktop computer) is possible if no smartphone or tablet is used. Insured individuals may grant access rights to a representative (eg, a family member). The authorized representative can view and manage the ePA on behalf of the insured person via the application. Initial access requires identity verification. Verification can be completed either via the electronic identity card in combination with a PIN or via the electronic health card in combination with a personal identification number (PIN). The PIN for the electronic health card must be requested from the health insurer. Issuance of the PIN requires prior identity verification, which can be conducted either in person at a branch office of the health insurer or through a postal identification procedure (PostIdent). In addition to the electronic health card, insured individuals may apply for a digital identity (GesundheitsID) through their health insurer. Following the application, authentication via electronic identity card or electronic health card and PIN is likewise required. After successful initial registration, simplified authentication methods may be used for subsequent logins, such as biometric procedures (eg, facial recognition), provided the device supports these functions. Available authentication options may vary depending on the device and operating system (Figure 1). The ePA is also established for children and adolescents. Until the age of 15 years, the ePA is managed by the parents or another legal guardian, who may also object to its use on behalf of the child. Upon reaching the age of 15 years, adolescents may independently manage their electronic patient record and may also exercise their own right to object [22].

**Figure 1:**
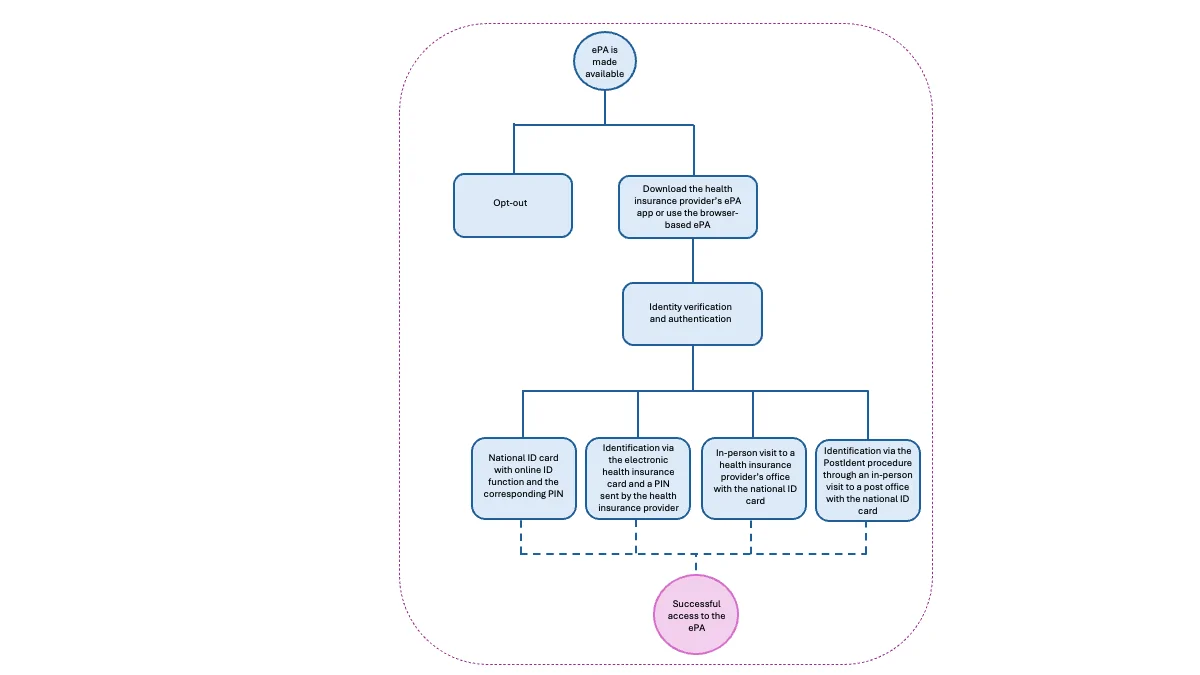
Depiction of the elektronische Patientenakte (ePA) access and authentication process (own illustration). PIN: personal identification number; PostIdent: postal identification procedure.

### Participants

Participants were recruited with the support of regional Associations of Statutory Health Insurance Physicians in Germany, including the Association of Statutory Health Insurance Physicians in Westphalia-Lippe, the Association of Statutory Health Insurance Physicians in Schleswig-Holstein, the Association of Statutory Health Insurance Physicians in Lower Saxony, and the Association of Statutory Health Insurance Physicians in Berlin. These associations contacted general practitioner (GP) practices within their respective regions and invited them to support the recruitment process. Participating GP practices received study flyers and written information materials describing the aim, procedures, and inclusion criteria of the study. The materials were made available to patients in the practices. Persons who were interested in participating were invited to contact the research team directly. Registration for study participation was possible via email or telephone. Participants were selected using purposive sampling [23]. Individuals were eligible to participate in the study if they met the following inclusion criteria:

- Legally insured under the German health insurance system
- Aged 18 years or older
- Experience as an active ePA user (defined as individuals who had successfully completed authentication and accessed the ePA through their insurer-provided application at least once) or passive ePA user (defined as individuals for whom an ePA had been created but who did not actively use the application)
- Intention to access the ePA through an insurer-provided application
- Willingness to participate in a qualitative interview

In total, 61 interviews were conducted within the broader ePA4all project [21]. All 61 interviews from the broader ePA4all dataset were screened for first-hand accounts of setup and authentication for insurer-provided ePA apps. Interviews were included if participants had personally attempted to access the ePA through an insurer-provided application and described at least 1 concrete action or event during this process. Based on this criterion, 23 interviews were selected for in-depth analysis. The remaining 38 interviews were excluded because participants had not personally attempted to access the ePA through an insurer-provided application or did not describe a concrete action or event during setup or authentication; no other exclusion reasons were applied. Of the 23 included participants, 21 were active users. The 2 passive users had initiated setup or authentication but abandoned the process before successfully accessing the ePA.

### Research Team and Reflexivity

The interview team had academic backgrounds in health sciences and sociology. SM and FM had extensive experience in conducting and analyzing qualitative interviews. All interviewers had prior research experience in digital health and the German ePA, which may have sensitized them to implementation-related barriers and challenges. To reflect on the potential influence of these prior perspectives, interpretations and coding decisions were discussed within the research team and documented in analytic memos. None of the interviewers had a prior personal or clinical relationship with the participants. Transcripts and findings were not returned to participants because participant validation was not included in the study design. Instead, the transparency and dependability of the interpretation were supported through independent coding by 2 researchers and subsequent consensus discussions.

### Data Collection

Data were collected through semistructured qualitative interviews. An interview guide was developed based on the study objectives and existing research on EHRs and was refined iteratively during data collection. The interview guide addressed experiences with the setup of and authentication for insurer-provided ePA apps, perceived barriers, and the immediate transition to initial use or nonuse after access (Multimedia Appendix 1). The interview guide was piloted with the first 3 participants, and no revisions were required because the questions were understood and elicited relevant accounts of setup and authentication. The interview guide therefore remained unchanged during subsequent data collection. Interviews were conducted by researchers (SM, FM, AN, MN, and FS) via telephone between August and December 2025. All interviews were conducted in German, audio-recorded with informed consent, and transcribed verbatim. Transcripts were anonymized prior to analysis. In addition, brief field notes were documented after each interview to capture contextual impressions and support interpretation of the interview situation; these notes were not included in the formal analysis. Saturation was assessed during the in-depth analysis of the 23 interviews included in the analytic subsample rather than used as a stopping criterion for data collection in the broader ePA4all study.

### Data Analysis

Qualitative analysis of the interviews was performed iteratively by SM and FM based on structured qualitative content analysis by Kuckartz [24] using MAXQDA Analytics Pro 2022 (version 22.1.0; Verbi GmbH). During initial familiarization with and coding of the broader dataset, all 61 interviews were screened for relevance to the present research question. The analytic subsample was selected before the subsequent in-depth analysis of the setup and authentication process. During initial coding of the 23 included interviews, recurring topics were grouped into inductively developed main categories and subcategories. The 5 process phases were developed from the coded material and were not predefined by the structure of the interview guide. The coding system was iteratively reviewed and revised by comparing coded passages within and across interviews. Category definitions, coding rules, and illustrative examples were documented in a codebook. Analytic decisions, revisions to the category system, and points discussed during consensus meetings were documented in analytic memos. Both researchers (SM and FM) independently coded all 23 interviews using the agreed coding system. Differences in coding or category interpretation were discussed until consensus was reached. Where necessary, category definitions were clarified and the relevant material was recoded. This procedure was intended to strengthen the transparency and dependability of the analysis.

In a second analytic step, recurring patterns of access barriers across the coded material were systematically compared. Beyond phase-specific challenges, the analysis focused on the relationships between successive phases of the access process. Through iterative comparison of cases and categories, we examined how difficulties arising in one phase affected participants’ progression through subsequent phases. Particular attention was paid to recurring sequences involving unclear requirements, increased coordination work, technical or administrative failure, interruption, repeated attempts, and disengagement. This second step resulted in a process-oriented interpretation of how barriers could interact and accumulate across the authentication pathway. The concept of authentication as an obligatory passage point served as a sensitizing perspective for examining how users, devices, applications, identification services, and administrative procedures had to be coordinated and how disruptions in one component affected the wider access process.

Within the analytic subsample, saturation was considered reached when the analysis of additional interviews produced no new process phases, categories, or relevant dimensions of the setup and authentication experience [25].

The data were analyzed in German. To present the findings, significant excerpts from the transcriptions were chosen as representative quotes. These quotes were translated into English and incorporated into the manuscript. The translated quotations were reviewed against the original German transcripts by bilingual members of the research team to ensure that their meaning and tone were retained. The manuscript was prepared in accordance with the COREQ (Consolidated Criteria for Reporting Qualitative Research; checklist provided in Multimedia Appendix 2) [26].

## Results

### Sociodemographic Characteristics

Of the 23 participants, 21 were active ePA users and 2 were passive ePA users. The 2 passive users included in the analysis had initiated setup or authentication but abandoned the process before successfully accessing the ePA. The mean age of the participants was 47.9 (SD 17.3; range 21-80) years. Most participants were male (male: n=14; female: n=9). The mean duration of the interviews was 35 (SD 8.3; range 18-50) minutes. The participants were insured with 11 different health insurance companies. Detailed characteristics of the study participants are shown in Table 1.

**Table 1:** Sample characteristics and ATI^a^ score (computed as the mean of ATI items; scale range 1-6; categorized as low: 1.00-2.66, moderate: 2.67-4.32, and high: 4.33-6.00).

ID	User type; age (years); sex	Population size (number of inhabitants), n	Chronic diseases	Operating system	ATI-S^b^ score and interpretation, mean (SD)
ePA_1a	Active; 30-39; male	3.8 million	No	iOS	5.5 (0.58); high
ePA_2a	Active; 20-29; male	60,000	No	iOS	5.0 (1.15); high
ePA_3a	Active; 30-39; female	12,000	Yes	iOS	5.5 (0.58); high
ePA_4a	Active; 50-59; male	12,000	No	Android	5.5 (0.58); high
ePA_5a	Active; 50-59; female	5000	Yes	Android	3.75 (0.96); moderate
ePA_6a	Active; 60-69; male	60,000	No	iOS	5.0 (1.15); high
ePA_7a	Active; 60-69; male	14,000	Yes	Android	6.0 (0.00); high
ePA_8a	Active; 60-69; female	6000	Yes	iOS	4.75 (0.96); high
ePA_9a	Active; 30-39; male	3.8 million	No	iOS	4.75 (1.50); high
ePA_10a	Active; 20-29; male	1 million	Yes	iOS	5.0 (1.15); high
ePA_11a	Active; 40-49; female	3.8 million	No	iOS	5.5 (0.58); high
ePA_12a	Active; 40-49; female	3.8 million	No	iOS	4.75 (0.96); high
ePA_13a	Active; 30-39; female	500,000	No	iOS	3.5 (0.58); moderate
ePA_14a	Active; 50-59; female	30,000	Yes	iOS	4.5 (S0.58); high
ePA_15a	Active; 80-89; male	1000	Yes	iOS	5.25 (0.50); high
ePA_16a	Active; 40-49; female	3000	Yes	iOS	4.5 (0.58); high
ePA_17a	Active; 30-39; male	550,000	No	iOS	5.0 (2.00); high
ePA_18a	Active; 60-69; male	26,000	No	Android	4.5 (1.73); high
ePA_19a	Active; 20-29; male	3.8 million	No	iOS	4.5 (1.73); high
ePA_20a	Active; 20-29; male	600,000	Yes	iOS	4.25 (0.96); high
ePA_21a	Active; 40-49; male	170,000	Yes	Android	5.5 (0.58); high
ePA_1p	Passive; 60-69; female	3.8 million	Yes	Android	3.5 (1.29); moderate
ePA_2p	Passive; 80-89; male	3.8 million	Yes	Android	2.5 (0.58); low

^a^ATI: Affinity for Technology Interaction.

^b^ATI-S: Affinity for Technology Interaction Short Scale.

The setup and authentication required to access the ePA through insurer-provided apps do not take place in a single step but unfold across several consecutive phases. Challenges rarely occur in isolation, but may interact and accumulate throughout the process. In the following, these barriers are traced along the individual process phases. Figure 2 visualizes the multistep access process of the ePA as a sequential process consisting of 5 inductively derived phases: orientation, technical entry, verification, interruption, and transition to initial use.

**Figure 2:**
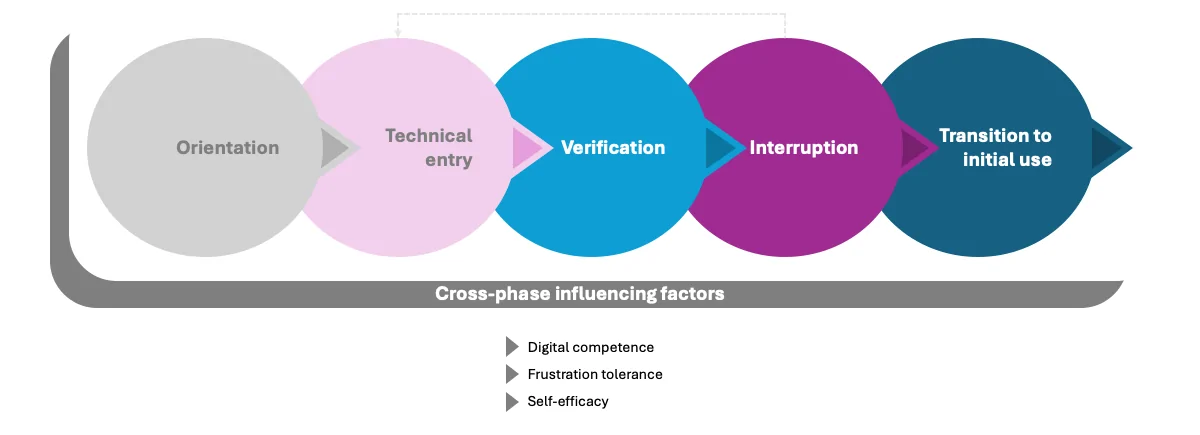
Summary of the experience of the elektronische Patientenakte (ePA) setup and usage process from the perspective of individuals with statutory health insurance.

### Phase 1: Orientation

In the first phase, barriers arise even before the actual registration begins, as potential users do not perceive the process as a clearly plannable and reliable sequence of steps but rather as an uncertain endeavor with unclear requirements and insufficient guidance. The available information is experienced as inconsistent and outdated.

Well, there is a video, and the video seems very outdated. (...) Apparently, the health insurance company did not update it.

ePA_13a_37_female

The orientation gap does not only concern the timeliness of information but also the coverage of concrete use cases. This becomes particularly evident in special situations, such as setting up the ePA application for children.

I had no idea how I was supposed to do that [install the ePA app for my child]. I simply didn’t know.

ePA_13a_37_female

As a result, entry into the process is not structured by a clear and comprehensible procedure but rather by an unsystematic search for relevant information, in which key courses of action remain unclear (eg, setting up the application for children or other persons). Consequently, the registration process often begins without a clear understanding of which steps are required in which order and what prerequisites need to be met.

### Phase 2: Technical Entry

The phase of technical entry is characterized by structural challenges. After downloading the ePA application, entry is experienced as a fragmented sequence of different applications, procedural paths, and decision points, the underlying logic of which is only partially comprehensible to users. The need to switch between various apps, identification options, and technical interfaces generates early feelings of overload and uncertainty about the next step. This experience is described as highly burdensome.

At that time, I was really somewhat overwhelmed by the whole process; I experienced it as really very labor-intensive.

ePA_12a_49_female

The metaphor of an “authentication jungle” illustrates the perception of a confusing and difficult-to-navigate system.

I just got into this authentication jungle.

ePA_1a_34_male

Entry into the system is experienced as a process in which users have to orient themselves independently, without being actively supported by the system architecture. This fragmentation does not only concern the number of steps but also their organizational and technical distribution. Entry does not take place via an integrated platform but through a combination of the main application, identification services, and, in some cases, separate service applications.

Yes, this whole registration process, right, with [name of health insurance company], you have to download two apps (...) and then you also have to open a second app. (...) Especially since you can’t even get into the patient record without opening the other app, so it’s basically duplicated.

ePA_7a_60_male

As a result, a high level of coordination is required from users right from the beginning, as they must relate various technical and administrative components to one another. A lack of transparency regarding which component fulfills which function intensifies the feeling of being caught in an opaque procedure whose logic is not immediately apparent.

In addition, technical access requirements act as independent barriers that can partially prevent entry altogether. Several participants report that the process failed due to unmet device requirements or security settings.

And then it said, you don’t have NFC on your device, you can’t use this device.

ePA_2p_80_male

Such requirements often appear unexpectedly and are usually neither transparently communicated in advance nor easily resolved in the short term, especially when they concern hardware-related or security-relevant settings. Access to the ePA thus depends on the availability of suitable end devices. Participants whose devices did not meet these requirements were unable to proceed with the access process using those devices.

At the same time, some participants report a smooth entry when technical requirements are met and prior experience is available. In these cases, entry is perceived as uncomplicated.

Well, everything went smoothly.

ePA_3a_36_female

However, these positive experiences are limited to specific constellations and do not represent a systematic characteristic of the procedure.

### Phase 3: Verification

Identity verification is not experienced as a single, clearly delineated step but as a complex sequence of multiple, partly redundant security measures, the internal logic of which is only partially comprehensible to users. Over the course of the process, various identification tools, passwords, PINs, one-time codes, and external verification services have to be coordinated. The parallel use of different security mechanisms increases cognitive load and leads to frustration.

And overall, there was this constant verification and logging in. And all these codes you get by email. It’s just a lot.

ePA_9a_35_male

At the same time, uncertainty arises as to whether the current step has been completed correctly and whether it will result in sustainable progress within the process. Identification is thus not experienced as a stable transition point but as a fragile intermediate state that can collapse at any time.

I felt like in the story of the hare and the hedgehog. You’re constantly being sent back and forth.

ePA_2p_80_male

This fragility becomes particularly evident in the frequently reported technical breakdowns during verification. Several participants describe that redirects to external identification services or internal verification modules do not function reliably.

Then, in that verification process, the page just didn’t load.

ePA_9a_35_male

In other cases, the process breaks down completely.

The process technically crashed for me, and then I basically had to do it several times.

ePA_17a_32_male

Such breakdowns not only terminate the current step but often lead to previously completed verification stages losing their validity. Users are therefore forced to repeat the entire identification process, regardless of how far they had already progressed. The associated uncertainty increases emotional pressure, as each additional attempt is accompanied by the expectation of renewed failure.

In addition to technical disruptions, administrative challenges also act as independent barriers in this phase, for example, due to inconsistencies in personal data.

That didn’t work either, because somewhere the addresses weren’t exactly the same.

ePA_19a_23_male

These administrative challenges are experienced as particularly burdensome, as they cannot be overcome through personal technical skills or increased effort.

### Phase 4: Interruption

Even after successfully completing individual steps, access to the ePA is often not enabled immediately but delayed due to postponed feedback, postal mailings, or time-limited activations. As a result, the registration process is not experienced as a continuous flow but as a sequence of separate steps that extend over several days or weeks.

It wasn’t just one day; it was several days, because I (...) still had to wait for an activation letter.

ePA_17a_32_male

These temporal interruptions force users to repeatedly resume the process. After each pause, they must reorient themselves, recall previously completed steps, and reconstruct the current status. Repeated interruptions have a direct impact on motivation. The initial willingness to invest effort into the setup diminishes over time when the process stretches across longer periods. The need to re-enter a complex technical procedure after days or weeks is perceived as an additional burden and often results in abandonment.

I also failed at some point during authentication and then I just gave up.

ePA_1a_34_male

In combination with the previously described technical and administrative barriers, these interruptions contribute substantially to the perception of the setup process as lengthy and disproportionately cumbersome.

I experienced that as extremely complicated (...). Very, very rigid.

ePA_17a_32_male

### Phase 5: Transition to Initial Use

Participants’ accounts also provided limited insight into what occurred immediately after successful setup and authentication. Formal access did not necessarily lead to initial or continued engagement with the respective ePA application. Despite completing the access process, some participants reported only sporadic or no subsequent use. In the transition to actual use, it becomes apparent that successfully completing registration and authentication does not automatically lead to sustainable integration of the ePA application into users’ everyday lives. Instead, the step from formal access authorization to actual, routinized use often fails to materialize. Despite overcoming multiple hurdles, participants report only sporadic or completely absent use of the application. For these participants, completion of setup marked the end of an effortful access process but not necessarily the beginning of regular application use.

A central reason for this lack of subsequent engagement was the limited perception of an immediate benefit. Many participants are unable to identify a clear added value after successful setup that would justify continued engagement with the ePA.

I installed it, but I never used it, because I didn’t see any benefit in it.

ePA_13a_37_female

The app was not always experienced as an integral part of medical care or personal health management but rather as an optional add-on whose immediate relevance was unclear. Consequently, successful access did not necessarily result in initial or continued engagement with the respective application.

### Cross-Phase Influencing Factors

Across all phases, participants drew on individual coping resources to navigate unclear instructions, coordinate multiple technical components, resolve unexpected problems, and resume interrupted procedures. These resources included digital competence, prior experience, persistence, and self-efficacy. This was explicitly reflected upon by participants.

If I didn’t have a certain persistence or digital affinity, I probably would have given up.

ePA_6a_67_male

The registration process was therefore often experienced as an individual problem-solving project without systematic support.

I basically muddled through it on my own.

ePA_12a_49_female

### Accumulation of Barriers Across the Access Process

Comparison across the 5 phases indicated that barriers could become consequential through their accumulation rather than through any single requirement. Unclear or outdated information during orientation meant that some participants entered the process without knowing which applications, documents, devices, or authentication procedures would be required. During technical entry and verification, they then had to coordinate multiple applications, identification services, passwords, PINs, and technical prerequisites. When technical failures, inconsistent administrative data, or postal procedures interrupted progress, previously completed steps sometimes had to be repeated. Each interruption therefore created additional coordination work and required participants to reorient themselves within the process.

These recurring sequences provide a process-oriented explanation of how access could become increasingly burdensome. Technical or administrative problems did not only affect the step in which they occurred; they could also prolong the pathway, require repeated attempts, and contribute to frustration or disengagement. Individual resources such as digital competence, prior experience, persistence, and self-efficacy helped participants navigate these demands but did not prevent technical or organizational failures. The findings therefore locate access difficulties in the alignment of user actions, technical components, and organizational procedures. When these components were not coherently connected, participants had to compensate through additional coordination and problem-solving work.

### Variation Across Insurers, Devices, and Authentication Pathways

Participants were insured with 11 different statutory health insurers and used both iOS and Android devices. Cross-case comparison indicated that the central process-related difficulties were not confined to a single insurer or operating system. Unclear guidance, coordination between multiple technical components, repeated verification steps, and interruptions were described across different application and device constellations. No consistent pattern indicating systematically greater difficulties with either iOS or Android was identified. Other barriers were conditional on the authentication route used. Near-field communication (NFC) capability was relevant when electronic identity cards or electronic health cards were used for authentication. The need to request a health card PIN or wait for an activation letter applied only to pathways requiring these credentials. Problems with redirects or switching between apps arose when external identification services or separate authentication apps were involved. Administrative inconsistencies, such as differences in address data, could prevent verification independently of device type. Because individual insurers were represented by only a small number of participants and authentication routes were not distributed systematically across the sample, the study was not designed to compare or rank specific insurers, apps, operating systems, or authentication methods. The findings therefore distinguish between broadly recurring and pathway-specific barriers but do not support conclusions about the relative performance of individual implementations.

## Discussion

### Principal Findings

This study examined how individuals with statutory health insurance experienced setup and authentication for insurer-provided ePA apps and which challenges affected successful access to the ePA. The results show that setting up the ePA is not perceived as a single, completed step but rather as a sequential process that spans several stages separated in terms of time and organization. The fifth phase, transition to initial use, should be interpreted as an immediate downstream consequence of the access process rather than as an analysis of sustained adoption or integration into everyday practice. Informational, technical, administrative, and temporal difficulties may interact across these stages, requiring users to repeatedly coordinate components, resolve problems, and resume interrupted procedures. The results are consistent with international studies on EHRs, which also report heterogeneous use and complex barriers to access [27].

At the beginning of the access process, participants encountered limitations related to incomplete, unclear, or outdated information. This finding is consistent with international research emphasizing the importance of information for access to patient-facing EHR systems [6,7,28]. Within the present process model, orientation was not merely a preliminary question of awareness. It influenced whether participants understood which applications, documents, technical requirements, and authentication steps would be required. Insufficient orientation could therefore increase the coordination work needed during technical entry and verification and contribute to the accumulation of burdens across subsequent phases.

The inductively identified phases indicate that challenges do not arise from isolated technical problems alone but rather from the interaction between technical systems, organizational structures, and user practices. The ePA registration process comprises several interrelated components: digital infrastructures (eg, applications, authentication, and identity services), organizational actors and procedures (eg, health insurers, identity verification processes, and postal delivery of access credentials), as well as the work required from users to understand, coordinate, and repeatedly navigate these components. When these components were not coherently aligned, difficulties could extend beyond the phase in which they initially occurred. Unclear requirements increased the coordination required during technical entry, technical or administrative failures interrupted verification, and waiting periods required users to resume the process after temporal gaps. Repeated attempts and prolonged effort could subsequently contribute to frustration and disengagement. The phases should therefore not be understood as independent categories or as a uniformly linear sequence, but as an interdependent process in which difficulties may accumulate and return users to earlier stages. These findings are consistent with insights from implementation research, which demonstrate that the successful introduction of digital technologies depends not only on technical functionality but, to a significant extent, on their organizational embedding and the provision of clear, comprehensible user guidance [29].

Importantly, substantial difficulties were also reported by participants with moderate or high affinity for technology. The findings therefore cannot be explained solely by limited digital competence. Participants’ knowledge, prior experience, self-efficacy, and persistence helped them navigate the process, but these resources could not compensate for incompatible devices, malfunctioning applications, inconsistent administrative data, or delayed activation procedures. Access should therefore be understood as emerging from the interaction between users’ available resources and the informational, procedural, technical, and organizational demands created by the access process. These findings can be situated within the eHealth Literacy Framework, which conceptualizes engagement with digital health as an interaction between individual capabilities and the characteristics of digital systems and services [30]. The present study does not propose an alternative or extended literacy framework. Rather, it provides a process-oriented account of how this interaction unfolds during setup and authentication. User resources influenced participants’ ability to interpret instructions, coordinate applications, solve problems, and resume interrupted procedures. At the same time, process design and technical and organizational conditions determined the demands they encountered and whether progression through the pathway was possible.

Conceptualizing authentication as a multistage obligatory passage point highlights that access depends on the successful alignment of users, devices, applications, identification services, health insurers, and administrative procedures [19]. The findings indicate that these components did not always operate as a coherent access arrangement. Participants had to bridge gaps between them by identifying the correct application, interpreting system feedback, coordinating authentication tools, resolving administrative inconsistencies, or resuming procedures after delays. Technical and administrative disruptions therefore became user-facing access barriers that required additional coordination and problem-solving work. Authentication should consequently be understood not as a neutral technical prerequisite but as a sociotechnical implementation stage that structures whether individuals can actively access a formally available national EHR infrastructure.

The findings raise equity-relevant concerns but do not provide direct evidence of differences in access between population groups. Participants described substantial demands related to digital competence, persistence, problem-solving, and access to compatible devices. Because the sample predominantly comprised motivated participants with moderate or high affinity for technology, most of whom ultimately accessed the ePA successfully, the study cannot determine how these demands affect groups with fewer resources. It is plausible that the identified requirements may be particularly consequential for individuals with fewer digital, material, linguistic, cognitive, or social resources. However, this represents an inference from the observed process demands rather than a direct empirical finding. This interpretation is consistent with international research showing that digital health technologies can reinforce disparities when effective access depends on unequally distributed resources [31-33]. Further research specifically sampling and comparing diverse population groups is needed to examine these equity implications.

### Practical Implications

The findings indicate that barriers to ePA access are embedded in the overall architecture and sequencing of the registration process rather than in isolated technical malfunctions. Implementation efforts should therefore focus on reducing cumulative burdens that emerge across stages. The practical implications were structured according to the 5 phases of the access process and the cross-phase factors identified in the analysis. For each phase, 1 priority measure was linked directly to the corresponding empirical finding. An additional cross-phase measure addresses participants’ reliance on individual coping resources throughout the pathway (Table 2).

**Table 2:** Practical implications to support access to the ePA^a^ application.

Process phase	Empirical finding	Practical implications
Phase 1: orientation	Participants began registration without a clear understanding of the required applications, documents, PINs^b^, device functions, or available authentication routes. Information was sometimes incomplete, outdated, or insufficient for specific situations.	Provide centralized, up-to-date, and pathway-specific guidance, including a preregistration checklist of all technical and administrative requirements.
Phase 2: technical entry	Switching between applications, identification services, and technical interfaces increased coordination work. Device requirements such as NFC^c^ capability were not always communicated before the process began.	Minimize transitions between applications and clearly communicate device and system requirements before users enter the authentication pathway.
Phase 3: verification	Multistep verification, technical failures, unclear feedback, and inconsistencies in administrative data prevented users from completing authentication or required repeated attempts.	Simplify verification procedures and provide actionable error messages that identify the problem and explain the next step or available support.
Phase 4: interruption	Postal procedures, waiting periods, and technical disruptions interrupted progression and required users to reconstruct or repeat previously completed steps.	Preserve completed steps and enable users to resume the process after interruptions, supported by clear progress and status information.
Phase 5: transition to initial use	Successful registration did not necessarily lead to initial or continued use when participants could not identify an immediate practical benefit.	Provide a brief postregistration orientation showing relevant initial use cases and the information or functions available in the ePA application.
Cross-phase factors	Across the pathway, participants relied on digital competence, prior experience, persistence, and self-efficacy to understand requirements, resolve problems, and resume interrupted procedures. The process was often experienced as an individual problem-solving task without systematic support.	Provide consistent and easily accessible support throughout the access pathway, including telephone or in-person assistance, to reduce reliance on individual coping resources.

^a^ePA: elektronische Patientenakte.

^b^PIN: personal identification number.

^c^NFC: near-field communication.

### Strength and Limitations

To the best of our knowledge, this is the first qualitative study to examine the setup and authentication process of the opt-out ePA in Germany from the perspective of people with statutory health insurance. The study provides detailed insights into process-related burdens that are often missed in survey-based research. The qualitative approach enabled the identification of cumulative barriers and their interaction across successive process phases and provided a nuanced understanding of how structural features of digital implementation influence access and the transition to initial use. At the same time, several limitations warrant consideration. The analysis is based on a purposively selected analytic subsample from a larger dataset, which limits the transferability of the findings beyond the study context. Participation was voluntary, and the sample predominantly comprised motivated participants with moderate or high affinity for technology. Moreover, 21 of the 23 participants had ultimately gained active access to the ePA application. The study may therefore underrepresent individuals who did not attempt registration, abandoned the process at an early stage, had limited digital experience, or lacked access to suitable devices or support. Consequently, the findings are well suited to demonstrating that substantial access difficulties can occur even among technologically confident and predominantly successful users, but they do not support conclusions about the prevalence of dropout or the experiences of socially, linguistically, cognitively, or digitally disadvantaged groups. The results are based on retrospective self-reports and may therefore be influenced by recall bias, particularly regarding the exact sequence of authentication steps, error messages, and waiting periods. The interviews captured participants’ subjective reconstruction of the access process rather than direct observations of their interactions with the applications. Furthermore, the study reflects experiences from an early phase of opt-out implementation; subsequent procedural or technical changes may alter user experiences over time. Because the interviews did not systematically examine use over an extended period, the findings do not support conclusions about sustained adoption or the long-term integration of the ePA into everyday health management. Since the interviews were conducted in 2025 and additional or different registration options have been introduced since then, the results reflect the registration procedures in effect at that time.

### Conclusion

This study explores access to the opt-out ePA as a multistage process rather than a single technical act. Obstacles may interact and accumulate in the areas of orientation, technical access, verification, interruption, and the transition to use, reflecting the interplay of infrastructure design, organizational processes, and individual coping resources. Formal availability therefore does not ensure straightforward access. More coherent process design, reduced fragmentation, and accessible support are needed to limit the extent to which successful access depends on individual coping resources. The equity implications of these process requirements should be examined in studies specifically designed to include and compare populations with different levels of digital, material, linguistic, cognitive, and social resources.

## Data Availability

The qualitative interview data generated and analyzed during this study are not publicly available because they contain sensitive information and may permit participant identification. Public sharing would not be compatible with the conditions of the ethical approval and participants’ informed consent. Requests concerning access to selected anonymized data may be directed to the corresponding author and will be considered subject to ethical, legal, and data protection requirements. For further questions regarding the reuse of data, please contact the corresponding author.

## Appendix

Multimedia Appendix 1 Interview guide.

Multimedia Appendix 2 COREQ (Consolidated Criteria for Reporting Qualitative Research) checklist.

## Abbreviations

COREQ: Consolidated Criteria for Reporting Qualitative Research
EHR: electronic health record
eID: electronic ID
ePA: elektronische Patientenakte
GesundheitsID: digital identity
GP: general practitioner
HCP: health care professional
NFC: near-field communication
OPP: obligatory passage point
PIN: personal identification number
PostIdent: postal identification procedure
