# Supplementary material for: Accessing Germany’s Electronic Patient Record Through Health Insurer–Provided Apps: Qualitative Interview Study
Source: JMIR Mhealth Uhealth. 2026 Sep 14;14:e95294. doi: 10.2196/95294 (PMC13620427; doi:10.2196/95294)
Supplement: Supplementary file 1 [file mhealth_v14i1e95294_app1.pdf]

Research Question: How do insured persons experience the registration process for the ePA and what obstacles influence successful access?			
Main Question / Narrative Prompt	Check Aspects	Follow-up Prompts and Probing Questions	Probing and Steering Questions
How did you experience the setup/installation of the electronic patient record (ePA)?	<u>Prerequisites and initial usage experiences</u><br>-Technical devices (smartphone, tablet)<br>-Support services (e.g., from relatives, health insurance provider)<br>-Authentication process<br>-Usability of the app/platform	How did the registration process proceed?<br><br>How was identity verification carried out?<br><br>Would you describe the process up to the first actual use as rather easy or rather burdensome? Please tell me about it.<br><br>Were there any technical or organizational hurdles during the initial use? What worked well?<br><br>Did you need or seek support, and if so, from whom?	Could you tell me more about that?<br>What happened next?<br>How did that feel for you?<br>How do you see that?<br>Could you elaborate on that, please?<br>Could you give an example?<br>What do you mean by that specifically?
