# Supplementary material for: Accessing Germany’s Electronic Patient Record Through Health Insurer–Provided Apps: Qualitative Interview Study
Source: JMIR Mhealth Uhealth. 2026 Sep 14;14:e95294. doi: 10.2196/95294 (PMC13620427; doi:10.2196/95294)
Supplement: Supplementary file 2 [file mhealth_v14i1e95294_app2.pdf]

Supplementary Material 2

Consolidated criteria for reporting qualitative studies (COREQ): 32-item checklist

Developed from:
Tong A, Sainsbury P, Craig J. Consolidated criteria for reporting qualitative research (COREQ): a 32-item checklist for interviews and focus groups. *International Journal for Quality in Health Care*. 2007. Volume 19, Number 6: pp. 349 – 357

Topic	Item No.	Guide questions/description	Reported on Page #
Domain 1: Research team and reflexivity			
Personal Characteristics			
Interviewer/facilitator	1	Which author/s conducted the interview or focus group?	11
Credentials	2	What were the researcher's credentials? E.g. PhD, MD	Author information, p 1
Occupation	3	What was their occupation at the time of the study?	Author affiliations, p 1
Gender	4	Was the researcher male or female?	Not reported
Experience and training	5	What experience or training did the researcher have?	10
Relationship with participants			
Relationship established	6	Was a relationship established prior to study commencement?	10
Participant knowledge of the interviewer	7	What did the participants know about the researcher? e.g. personal goals, reasons for doing the research	Study purpose: p 7
Interviewer characteristics	8	What characteristics were reported about the inter viewer/facilitator? e.g. Bias, assumptions, reasons and interests in the research topic	10
Domain 2: study design			
Theoretical framework			
Methodological orientation and Theory	9	What methodological orientation was stated to underpin the study? e.g. grounded theory, discourse analysis, ethnography, phenomenology, content analysis	11-12
Participant selection			
Sampling	10	How were participants selected? e.g. purposive, convenience, consecutive, snowball	9-10
Method of approach	11	How were participants approached? e.g. face-to-face, telephone, mail, email	9
Sample size	12	How many participants were in the study?	10, 13

Non-participation	13	How many people refused to participate or dropped out? Reasons?	Not available (open recruitment via study flyers)
Setting			
Setting of data collection	14	Where was the data collected? e.g. home, clinic, workplace	11
Presence of non-participants	15	Was anyone else present besides the participants and researchers?	Not reported
Description of sample	16	What are the important characteristics of the sample? e.g. demographic data, date	13
Data collection			
Interview guide	17	Were questions, prompts, guides provided by the authors? Was it pilot tested?	11; Supplementary Material 1
Repeat interviews	18	Were repeat interviews carried out? If yes, how many?	Not reported
Audio/visual recording	19	Did the research use audio or visual recording to collect the data?	11
Field notes	20	Were field notes made during and/or after the interview or focus group?	11
Duration	21	What was the duration of the interviews or focus group?	13
Data saturation	22	Was data saturation discussed?	11-12
Transcripts returned	23	Were transcripts returned to participants for comment and/or correction?	10
Domain 3: analysis and findings			
Data analysis			
Number of data coders	24	How many data coders coded the data?	11
Description of the coding tree	25	Did authors provide a description of the coding tree?	14-21; Figure 2 (p 14)
Derivation of themes	26	Were themes identified in advance or derived from the data?	11-12
Software	27	What software, if applicable, was used to manage the data?	11
Participant checking	28	Did participants provide feedback on the findings?	10
Reporting			
Quotations presented	29	Were participant quotations presented to illustrate the themes/findings? Was each quotation identified? e.g. participant number	15-19
Data and findings consistent	30	Was there consistency between the data presented and the findings?	14-21
Clarity of major themes	31	Were major themes clearly presented in the findings?	14-21
Clarity of minor themes	32	Is there a description of diverse cases or discussion of minor themes?	16, 18, 21
